# Acknowledgment of *Ad Hoc* Reviewers

**DOI:** 10.1128/mSystems.00298-18

**Published:** 2018-12-26

**Authors:** Jack A. Gilbert

**Affiliations:** a University of Chicago, Chicago, Illinois, USA

## EDITORIAL

We are coming to the end of 2018, which has seen *mSystems* become a recognized and prestigious venue for microbial systems biology studies. That would not have been possible had it not been for the scientists who have dedicated their time to supporting the fair and unbiased review of the science presented in *mSystems*. We are indebted to the many people who have contributed to article reviews over the last 3 years. What you all have sacrificed in time has been repaid in furthering the pursuit of knowledge in a format that is freely accessible to the whole world.

Stephen T. Abedon
Jonathan Miles Adams
Mark D. Adams
Sarah Ades
Pamela R. F. Adkins
Evelien M. Adriaenssens
Vinayak Agarwal
Brian M. M. Ahmer
Alexander Aksenov
Md Tauqeer Alam
Mads Albertsen
Pietro Alifano
Robert Allake
Raul A. Almeida
Eric Altermann
Katherine R. Amato
Anthony Amend
Amnon Amir
Karthik Anantharaman
Jamila Anba-Mondoloni
Cheryl P. Andam
Heidi M. Andersen
Rika Anderson
Raul Andino
Largus T. Angenent
Kym S. Antonation
Ayako Aoki-Yoshida
Cristian Apetrei
Michael A. Apicella
Hector Arguello-Rodriguez
Masanori Arita
Jean Armengaud
Magnus Ø. Arntzen
Mario Arrieta-Ortiz
Graeme Trevor Attwood
Jennifer Auchtung
Julian Avila Pacheco
Martin Iain Bahl
Abhay Bajaj
Brett J. Baker
Matthew G. Bakker
Chandra Shekhar Bakshi
David A. Baltrus
Carlos Barajas
Bridget Barker
Stephen Barnes
Francisco Barona-Gomez
Jeremy J. Barr
Jeffrey E. Barrick
Amanda Barry
Douglas H. Bartlett
Anke Becker
Sylvia Becker-Dreps
Eric Daniel Becraft
Peter Trip Beernink
Marcel A. Behr
Gordon Morse Bennett
Isabelle Benoit
Gabriele Berg
Ivan Berg
Hans C. Bernstein
Claire Bertelli
Stefan Bertilsson
Erin Bertrand
Wolfgang Beyer
Shantanu Bhatt
Kyle Bibby
Elisabeth M. Bik
Steven Biller
Fabrice Vincent Biot
Johannes Björk
Johanna Björkroth
Jill R. Blankenship
Steve Blazewicz
Ran Blekhman
Robert M. Blumenthal
Gregory Bokinsky
Nicholas Andrew Bokulich
Tobias Bollenbach
Gregory Bonito
Richard Bonneau
Erika Bonsaglia
Rafaella C. Bonugli-Santos
Anna Both
Brian Bothner
David G. Bourne
John P. Bowman
Sean F. Brady
Axel A. Brakhage
Jonathan Braun
Asker Daniel Brejnrod
Orianna Bretschger
Tess Elizabeth Brewer
Lazzaro Brian
David S. Briskey
Brandon Brooks
Paul Brooks
C. Titus Brown
Daniel R. Brown
Jeremy C. Brownlie
James E. Bruce
Donald A. Bryant
Samuel Joseph Bryson
Nicole R. Buan
Alison Buchan
Michael J. Buchmeier
Carmen Buchrieser
Sara Ashley Bumgardner
Adam R. Burns
Brendan Paul Burns
Frederic D. Bushman
Kyle C. Cady
Ben Callahan
Benjamin J. Callahan
Stephen J. Callister
Andres Mauricio Caraballo Rodriguez
Valerie Jean Carabetta
Paul Carini
Erin Carlson
Ross P. Carlson
Gilda Carvalho
Magali Chabé
Woo-Suk Chang
Michael S. Chaussee
Jun Chen
Tingtao Chen
Lei Cheng
Leonid Chindelevitch
Peter Chivers
Sidharth Chopra
Joseph Alexander Christie-Oleza
Geremy C. Clair
Tom Clavel
Luca Cocolin
David A. Coil
Andre M. Comeau
John Common
Laurie E. Comstock
Jonathan Conway
Tyrrell Conway
Vaughn S. Cooper
Otto X. Cordero
Elizabeth K. Costello
Natacha Couto
Don A. Cowan
Michael Cox
Katharine Coyte
John Cryan
Catalina Cuellar-Gempeler
Julia Cui
Christina Cuomo
Dennis G. Cvitkovitch
Christiane Dahl
Colin Dale
Remus Dame
Jeffrey L. Dangl
F. Darfeuille
Julian E. Davies
James Davis
Stacy L. Deblasio
Valérie De Crécy-Lagard
Michael Deem
Carlotta De Filippo
Nicholas R. De Lay
Celine Delbes-Paus
Edward F. Delong
Ye Deng
Muriel Derrien
Petra Dersch
Mahesh Desai
Adam M. Deutschbauer
Ken Dewar
Gregory J. Dick
Christian Diener
Dirk P. Dittmer
Jeremy A. Dodsworth
Claudio Donati
Yiran Dong
Mohamed S. Donia
Ian Donohue
Angela E. Douglas
Gavin M. Douglas
Jaquelin P. Dudley
Tim J. Dumonceaux
John Dunbar
Katherine R. Duncan
Taylor Dunivin
Ashlee M. Earl
Brian Joseph Eddie
Laura Eme
Bruce D. Erickson
Cassandra Lane Ettinger
Amandine Everard
Vanessa Ezenwa
Andrew James Fabich
Liam J. Fanning
Karoline Faust
Gabriel da Rocha Fernandes
Lucia Fernandez
Noah Fierer
Omri M. Finkel
Nahuel Fittipaldi
Erik K. Flemington
Harry J. Flint
Lucile M. Floeter-Winter
Yuriy Fofanov
Edan Foley
Marco Fondi
Joseph Che Forbi
Adriana Forero
Kristoffer Forslund
Samuel Charles Forster
Konrad Ulrich Förstner
Paul Forsythe
Jamie S. Foster
Zachary Foster
Fiona Fouhy
Miguel Frada
Pedro Frade
Pilar Francino
Eelco Franz
Sebastian Fraune
Stephen J. Free
Katie Freel
Pengcheng Fu
Jed Fuhrman
Tamas Gaal
Mihaela Gadjeva
Francesca Gaggia
Marco Galardini
Jinshan Gao
José Luis García
Jeffrey G. Gardner
Daniel Garrido
Caroline Attardo Genco
C. R. Robert George
Laurel J. Gershwin
Andrew Gewirtz
Raad Gharaibeh
Paolo Ghensi
Travis Gibson
Laura Glendinning
Gregory B. Gloor
Jon D. Goguen
Shan Goh
Susan S. Golden
Maria Adelaida Gomez
David J. Gonzalez
Andrew Goodman
Uri Gophna
Stephen V. Gordon
David C. Grainger
Jeffrey A. Gralnick
Leigh Greathouse
Stefan J. Green
Julian Griffin
Christopher A. Gulvik
Michelle Gwinn-Giglio
Elaine M. Haase
Markus Haber
Murray Hackett
Martin W. Hahn
Mehrdad Hajibabaei
Lindsay J. Hall
Nafiz Hamid
Robert E. W. Hancock
Kim M. Handley
Matthew J. Harke
Joe Harrison
Bernhard Haubold
Keith Hazelton
Ian Head
Brian P. Hedlund
Matthias Heinemann
Michael Henson
Ute Hentschel
Malte Herold
Anat A. Herskovits
Robert L. Hettich
Thomas Hitch
William E. Holben
Georgina Louise Hold
Johannes Holert
Emily B. Hollister
Matthias Horn
Jianzhong Hu
Sarah K. Hu
Shi Huang
Judith M. Hübschen
Laura A. Hug
Eili Huhtamo
Bruce A. Hungate
Dana E. Hunt
C. Neil Hunter
Daniel Huson
Embriette R. Hyde
Tadayuki Iwase
Jacques Izard
Jonathan L. Jacobs
Crystal Jaing
Michael Janech
Ian B. Jeffery
Poul Erik Jensen
Patricio Jeraldo
Ding Jun Jin
Seth G. John
Kay O. Johswich
Ian Joint
Kathryn M. Jones
Kirsten Jung
Nadeem O. Kaakoush
Krishna (Rani) Kalari
Vipin Chandra Kalia
Marina G. Kalyuzhnaya
Taro Kamagaki
Dae Wook Kang
Wei-Chun Kao
Wojciech Karlowski
Purna Chandra Kashyap
Anne-Kristin Kaster
Elena Kazamia
Sean Kearney
Scott T. Kelley
Colleen Kellogg
Scott Kenney
Saeed Khan
Khashayarsha Khazaie
Mary L. Killian
Minsuk Kim
Pan-Jun Kim
Benjamin C. Kirkup
Todd Kitten
Jonathan Klassen
Silke Klee
Vanja Klepac-Ceraj
Dan Knights
Kevin Kohl
Konstantinos T. Konstantinidis
Eugene V. Koonin
Omry Koren
Nicole Marie Koropatkin
David Koslicki
Aleksandar Kostic
Petia Kovatcheva
Jens H. Kuhn
Prashant Kumar
Roshan Kumar
Deepak Kumaresan
Elizabeth M. Kutter
Brandon LaBumbard
Douglas J. LaCount
Isabelle Laforest-Lapointe
Leo Lahti
Michael Lammers
Amy Lane
Jenna M. Lang
Pascal Lapierre
Adi Lavy
Vladimir Lazarevic
Sarah Lebeer
Yoann LeBreton
Fredrick J. Lee
Kyongbum Lee
Philippe Claude Lefebvre
Jose Lemos
Vanessa Leone
Sabine Leroy
Huiying Li
Tami Lieberman
Gipsi Lima Mendez
Kai-Shu Ling
Pushpinder Litt
Nan Liu
Richard Longnecker
Cedric Lood
Stilianos Louca
Petra Louis
Claude Loverdo
Kun Lu
Michael Lynch
Boris Macek
Barbara J. MacGregor
John MacSharry
Juliette Madan
Stefania Magnusdottir
Finlay Maguire
Frédéric Mahé
Vladimir Makarenkov
Thulani P. Makhalanyane
Meenakshi Malik
Elizabeth Mallott
Ohad Manor
Costas D. Maranas
Maria L. Marco
Angela Marcobal
Jeffrey Marlow
Natalie Marshall
Rebeca Martin
Adam Martiny
Bernd Masepohl
Andrea Masotti
Jessica R. McCann
Ryan McClure
Beth McCormick
John McCutcheon
Jason E. McDermott
Daniel McDonald
Andrew McDowell
Michael J. McInerney
Luke McKay
Jeffrey Scott McLean
Katherine McMahon
Alan McNally
Patrick J. McNamara
James F. Meadow
Marnix H. Medema
Monica Medina
Conor Meehan
Jay Mellies
Alexey V. Melnik
Bodo Melnik
Akram Mendez
Alessio Mengoni
Shiri Meshner
Ilhem Messaoudi
Jessica Metcalf
Thomas Metz
Kim Milferstedt
Andrew D. Millard
Shujiro Minami
Tim Miyashiro
Jennifer M. Mobberley
Andrew Moeller
Debasisa Mohanty
Hosein Mohimani
Jonathan Monk
Christophe Monnet
Lisa Moore
Nathaniel John Moorman
Charles P. Moran
Mary Ann Moran
Maria Aparecida Scatamburlo Moreira
Xochitl C. Morgan
Jenna Morgan-Lang
Andrey Morgun
Cindy E. Morris
J. Jeffrey Morris
Robert M. Morris
Elke Mühlberger
Maitreyee Mukherjee
Martha H. Mulks
Alvaro Munoz
Vivek Mutalik
Hiroaki Naka
Gerard J. Nau
Lama Nazzal
Julia Neilson
William C. Nelson
Camilla Lothe Nesbo
Markus Nett
Terry Fei Fan Ng
Nancy N. Nichols
Jens Nielsen
Jeppe Lund Nielsen
Olivia D. Nigro
Hiroshi Nikaido
Aleksandra Nita-Lazar
Krishna Niyogi
Cecilia Noecker
Kenneth M. Noll
Torbjörn Norén
Howard Ochman
Mary O'Connell-Motherway
Tadgh O'Croinin
Marco Rinaldo Oggioni
Stephen G. Oliver
Huub J. M. Op Den Camp
Aharon Oren
Hiroyuki Oshiumi
Elizabeth A. Ottesen
Abdullah Ozer
Olga Ozoline
Keith Paarporn
Oleg Paliy
Brent Palmer
Robert J. Palmer, Jr.
Chongle Pan
Laura Parfrey
John Parkinson
Kiran R. Patil
Vaishnavi Pattabiraman
Andrew D. Patterson
Joseph N. Paulson
Christopher Peacock
Shyamal D. Peddada
Beatriz Penalver
John Penders
Bingyin Peng
Charles Pepe-Ranney
Matthew Perisin
Jakob Pernthaler
Hannes Peter
Jason Peters
Stefan Pfeiffer
Vanessa Phelan
Jo Philips
Caroline C. Philpott
Pascal Pineau
Ameet J. Pinto
James M. Pipas
German Plata
Angela C. Poole
Phillip B. Pope
Alexa A. Pragman
Om Prakash
Michael B. Prentice
Lance B. Price
Morgan N. Price
Nathan Price
Sladjana Prisic
Catherine Putonti
Meng Qi
Jianming Qiu
Yuanyuan Qu
Zhe-Xue Quan
Robert Andrew Quinn
Elisabeth A. Raleigh
Srinivasan Ramachandran
Matthew Mark Ramsey
Christopher Rao
Philip N. Rather
Frederic Raymond
Gregor Reid
Pierre Renault
Francis Repoila
Scott Rice
Jason M. Ridlon
Lionel Rigottier-Gois
Daina L. Ringus
Judith Risse
Adam Rivers
Michael Scott Robeson
Dmitry A. Rodionov
Geraint B. Rogers
David A. Rosen
Ida Rosenkrands
Johannes Rousk
Simon Roux
Taniya Roy Chowdhury
Ben Rubin
Steven Rutherford
Ratul Saikia
Nina R. Salama
Hassan Samee
Alvaro Sanchez
Jon Sanders
Álvaro San Millan
Beng San Yeoh
Pauline Deirdre Scanlan
Patrick D. Schloss
Thomas Mitchell Schmidt
Lynn Schriml
Thomas Schweder
Herbert P. Schweizer
Henning Seedorf
Anna Maria Seekatz
Alecia Septer
Reed S. Shabman
William M. Shafer
Rushina Shah
Jason W. Shapiro
Om Prakash Sharma
Laura Jayne Sherrard
Rafael Silva-Rocha
Justin D. Silverman
Pallavi Singh
Yogendra Singh
Rohita Sinha
Lindsey M. Solden
Abraham L. Sonenshein
Se Jin Song
Justin Sonnenburg
Jörg Soppa
Diana Sousa
Vanessa Sperandio
Daan R. Speth
Joseph Evan Spraker
Stefan Spring
Eric V. Stabb
Pierre Stallforth
Lisa Y. Stein
Ralf Steuer
Chris Stewart
O. Colin Stine
Ulrich Stingl
Daniel Stoebel
Xiaoquan Su
Garret Suen
Matthew Sullivan
Fengzhu Sun
Jun Sun
Ming Sun
Shinichi Sunagawa
Michael G. Surette
Michiko E. Taga
Ilias Tagkopoulos
Guylaine Talbot
Régine Talon
Hideyuki Tamaki
Ming Tan
Patrick Tang
Gerald William Tannock
Suvi Taponen
Mike Taylor
Robert Thacker
Casey Theriot
Nicholas R. Thomson
Tamas Torok
Susannah Green Tringe
Pankaj Trivedi
Tommy Tsan-Yuk Lam
Tamir Tuller
Benjamin John Tully
Jenny Tung
Kieran Tuohly
Hanne L. P. Tytgat
Mario Uchimiya
Hidetoshi Urakawa
Tadasu Urashima
Sergio Uzzau
Juan José Valdez-Alarcón
Jairam K. P. Vanamala
Lia van der Hoek
Justin van der Hooft
Daniel van der Lelie
David van Duin
Geertje van Keulen
Tricia A. Van Laar
Gary Vanzin
Patricia Sampaio Tavares Veras
Kevin Verstrepen
Emily Vogtmann
William G. Wade
Irene Wagner-Dobler
Matthew K. Waldor
Levi Waldron
Alan W. Walker
Matthew Wallenstein
William Anton Walters
Chao-Min Wang
Guanghua Wang
Pauline W. Wang
Christopher Ward
Doyle Ward
Alex D. Washburne
Kenneth Wasmund
Mick Watson
Grzegorz Wegrzyn
Pamela Weisenhorn
Hartmut Wekerle
George F. Wells
Michiel Wels
Jeffrey Werner
Laura Weyrich
William B. Whitman
Shannon Whitmer
Giovanni Widmer
Sivaramesh Wigneshweraraj
Annegret Wilde
Michael James Wilkins
Benjamin P. Willing
Amy Willis
Jennifer R. Wilson
Kathryn Winglee
Benjamin E. Wolfe
Ben J. Woodcroft
Jeffrey A. Woods
Colin Worby
Aaron T. Wright
Erik S. Wright
Gerard D. Wright
Kelly Wrighton
Hao Wu
Michael Wu
Joao Xavier
Zhenjiang Zech Xu
Luying Xun
Jane Y. Yang
Stephanie A. Yarwood
Alyson Yee
Pelin Yilmaz
Yanbin Yin
Sang Sun Yoon
Mengting Yuan
Joseph P. Zackular
Elena Zaikova
Jin Zeng
Karsten Zengler
Kai Zhang
Lixin Zhang
Meiling Zhang
Wenjun Zhang
Haitao Zhao
Liang Zhao
Yingming Zhao
Huaijun Zhou
Jizhong Zhou
Luchang Zhu
Erik R. Zinser
Gang Zou
Peter Zuber

